# Virulence mismatches in index hosts shape the outcomes of cross-species transmission

**DOI:** 10.1073/pnas.2006778117

**Published:** 2020-10-29

**Authors:** Nardus Mollentze, Daniel G. Streicker, Pablo R. Murcia, Katie Hampson, Roman Biek

**Affiliations:** ^a^Institute of Biodiversity, Animal Health and Comparative Medicine, University of Glasgow, Glasgow G12 8QQ, United Kingdom;; ^b^Medical Research Council–University of Glasgow Centre for Virus Research, Glasgow G61 1QH, United Kingdom

**Keywords:** cross-species transmission, virulence, virus, rabies

## Abstract

Whether a pathogen entering a new host species results in a single infection or in onward transmission, and potentially an outbreak, depends upon the progression of infection in the index case. Although index infections are rarely observable in nature, experimental inoculations of pathogens into novel host species provide a rich and largely unexploited data source for meta-analyses to identify the host and pathogen determinants of variability in infection outcomes. We analyzed the progressions of 514 experimental cross-species inoculations of rabies virus, a widespread zoonosis which in nature exhibits both dead-end infections and varying levels of sustained transmission in novel hosts. Inoculations originating from bats rather than carnivores, and from warmer- to cooler-bodied species caused infections with shorter incubation periods that were associated with diminished virus excretion. Inoculations between distantly related hosts tended to result in shorter clinical disease periods, which are also expected to impede onward transmission. All effects were modulated by infection dose. Taken together, these results suggest that as host species become more dissimilar, increased virulence might act as a limiting factor preventing onward transmission. These results can explain observed constraints on rabies virus host shifts, describe a previously unrecognized role of host body temperature, and provide a potential explanation for host shifts being less likely between genetically distant species. More generally, our study highlights meta-analyses of experimental infections as a tractable approach to quantify the complex interactions between virus, reservoir, and novel host that shape the outcome of cross-species transmission.

## Significance

Emerging disease epidemics often result from a pathogen establishing transmission in a novel host species. However, for reasons that remain poorly understood, most cross-species transmissions fail to establish in the newly infected species. Examining experimental cross-species inoculations of rabies virus, we show that host and viral factors predict differences in disease progression in ways that are expected to impact the likelihood of onward transmission. Disease progression was accelerated and virus excretion decreased when the reservoir and novel host were physiologically or genetically more dissimilar. These insights may help to explain and predict host shifts in rabies and other zoonotic viruses and highlight meta-analyses of experimental inoculation data as a powerful and generalizable approach for understanding the dynamics of index infections.

Cross-species transmission is an important source of emerging and endemic disease. Viruses such as West Nile virus, rabies virus, and Lassa virus cause tens of thousands of human infections annually through transmission from animal reservoirs (1–3). Cross-species transmission is also the first step toward host shifts, where pathogens establish transmission cycles in novel hosts (4). While the broader-scale epidemiological and ecological factors driving cross-species transmission are beginning to be understood (reviewed in refs. 5–7), we remain unable to anticipate whether cross-species transmission will cause “dead-end” infections or transmit onward. Infection dynamics at the cross-species interface, specifically the probability of infection given exposure and the progression of index infections in novel hosts, are generally unobservable in nature. This is a crucial gap given that the outcomes of cross-species infections have profound implications for host shifts and disease emergence.

Cross-infection studies, in which viruses from a natural reservoir are experimentally inoculated into novel host species, provide a rare view into the dynamics of index infections. Since the dose, route, timing, and origin of viral exposure are known, these factors can be controlled for to identify the biological and evolutionary rules that govern the outcomes of cross-species transmission. We focus on *Rabies lyssavirus* (family *Rhabdoviridae*) as a model pathogen for understanding cross-species transmission (8). Rabies virus is a primarily bite-transmitted zoonotic RNA virus that infects all mammals and, untreated, has the highest case fatality ratio of any viral disease (9, 10). Rabies virus naturally infects multiple carnivore and bat species, which each perpetuate species-specific maintenance cycles (11). Although most cross-species transmission events do not lead to onward transmission, each maintenance cycle represents a rare past cross-species transmission event that established transmission in a novel host. Dead-end cross-species transmissions and historical host shifts are detectable in rabies virus phylogenies, and epidemiological surveillance reveals that nascent host shifts remain commonplace (11–13). As such, rabies virus exhibits extensive variation in the epidemiological outcomes of cross-species transmission. Here, we exploit cross-infection studies conducted over several decades, in which diverse mammalian species were inoculated with rabies viruses of bat and carnivore origin, to investigate the individual-level outcomes of index infections.

The potential for onward transmission of rabies virus is likely to depend on the incubation period (from bite to the appearance of clinical signs) and the duration of clinical signs prior to death (here, the clinical period) of infected hosts. Longer incubation periods are associated with greater distribution of virus through the central nervous system (14), spread to a wider range of tissues (15), and higher virus titers in the salivary glands, all of which should facilitate onward transmission (16). Conversely, faster progression of infection has been associated with lower virus excretion, and in extreme cases animals die before the virus reaches the salivary glands, making transmission highly unlikely (14–17). Further, the clinical period of rabies coincides with the period of greatest infectivity, when excretion of virus in the saliva often coincides with clinical signs such as aggression which promote transmission (18). Testing for shifts in incubation and clinical period durations, and in the amount of virus excreted, allows us to examine what plausible factors may constrain onward transmission of rabies in index hosts following cross-species transmission.

**Fig. 1.**
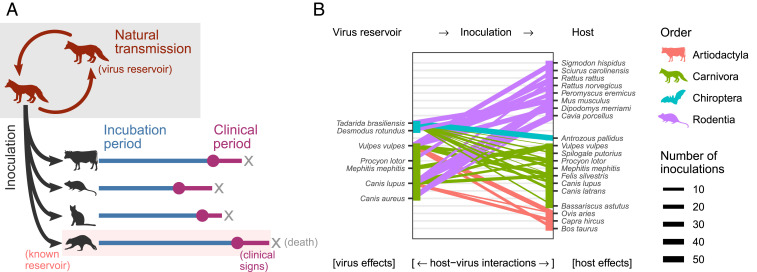
Experimental setup and data analyzed in this study. (*A*) Data were collected from published experiments in which naturally circulating rabies virus isolates were inoculated into heterologous species. The delay between inoculation and the appearance of clinical signs (the incubation period) and the delay between clinical signs first appearing and death (the clinical period) was recorded for individual animals. (*B*) Species combinations for which data were available. Colored lines connect observed reservoir-inoculated species pairs, with line thickness indicating the number of inoculations providing data on at least one of the variables analyzed (incubation period, clinical period, or salivary gland virus titer). Three reservoir species (*Canis aureus*, *Tadarida brasiliensis*, and *Desmodus rotundus*) received no inoculations which met our inclusion criteria.

Based on previous work on rabies virus and in other host–pathogen systems, several mechanisms are hypothesized to influence infection dynamics and the outcome of cross-species transmission (Fig. 1):

1) Features of exposed host species (host effects), irrespective of the infecting virus. For example, larger-bodied species may be more resistant to infection and thus require either higher infectious doses or a longer period of virus replication before symptoms become apparent. More generally, evolutionarily conserved similarities in host physiology mean that groups of related taxa might have similar susceptibility or clinical outcomes of infection (19).
2) Features inherent to the virus lineage involved (virus effects), irrespective of the infected host, likely due to adaptation of individual lineages to reservoir host species. Although rabies virus has shifted multiple times within and between bats and carnivores, only a handful of amino acid changes have been linked to host adaptation (12, 20, 21). Relatively little is known about how these amino acid changes contribute to infection phenotypes (21). Further, key differences in disease presentation between rabies viruses adapted to bats and those adapted to carnivores have been noted in humans, although it remains unclear whether this is a feature of the virus or due to differing routes of exposure (22).
3) Host–virus interactions. Both initial cross-species transmission and successful establishment occur most often between closely related hosts, often referred to as the phylogenetic distance effect (13, 19, 23, 24). However, the mechanisms underlying this pattern remain obscure. Mammals also exhibit considerable variability in physiological features that are only moderately constrained by phylogenetic relatedness, such as body temperature (25). This may create distantly related pairs of reservoir and novel host species which nevertheless share key physiological features affecting disease outcome (26), a potential explanation for the occurrence of host shifts over wide phylogenetic scales.

Here, we test these hypotheses by conducting a meta-analysis of individual-level data from 514 published experimental cross-species infections involving rabies virus. We show that features of the virus and of the inoculated host species interact with the initial conditions of exposure to influence the outcome of cross-species transmission in ways expected to affect the likelihood of onward transmission in the novel host species.

## Results

Our meta-analysis of cross-species inoculation experiments recorded results from 65 experiments in total. In agreement with observations from natural infections (27, 28), not all inoculations resulted in clinical rabies. Only 1,054 (63%) of the 1,672 inoculated animals for which any data were available developed rabies during the observation periods of the included experiments. The proportion of animals which developed rabies was similar among both within-species (525/822) and cross-species inoculations (529/850).

All analyses below were performed on data from cross-species inoculations only. A total of 30 experiments provided cross-species inoculation data for at least one of the three outcome measures of interest: the duration of the incubation period (*n* = 443 inoculations) and clinical period (*n* = 178) and the amount of virus excreted (*n* = 278). These experiments involved 20 mammal species (in the orders Carnivora, Chiroptera, Cetartiodactyla, and Rodentia), inoculated with 39 unique inocula from seven reservoir species in the orders Carnivora and Chiroptera (Fig. 1 and Table 1). The experiments analyzed were published in 23 publications between 1958 and 2013.

**Table 1.** Number of observations and hierarchical structure of data available from experimental cross-species inoculations involving rabies virus

	Incubation period	Clinical period	Virus titer in salivary glands
Individual inoculations (sample size)	443	178	278
Publications	19	15	16
Experiments	25	20	20
Inocula	35	18	22
Source taxonomic orders/species	2/7	2/4	2/4
Inoculated taxonomic orders/species	4/19	4/13	4/18
Source-inoculated species combinations	30	20	25

Inocula refers to the different virus isolates used; publications might contain multiple discrete experiments conducted by the same research group.

### Incubation Period.

The time period between inoculation and the appearance of symptoms was highly variable, with a median duration of 15 d. While incubation periods ranged between 4 and 141 d, 95% lasted ≤28 d (all estimates based on a nonparametric Kaplan–Meier fit to the censored event times).

**Fig. 2.**
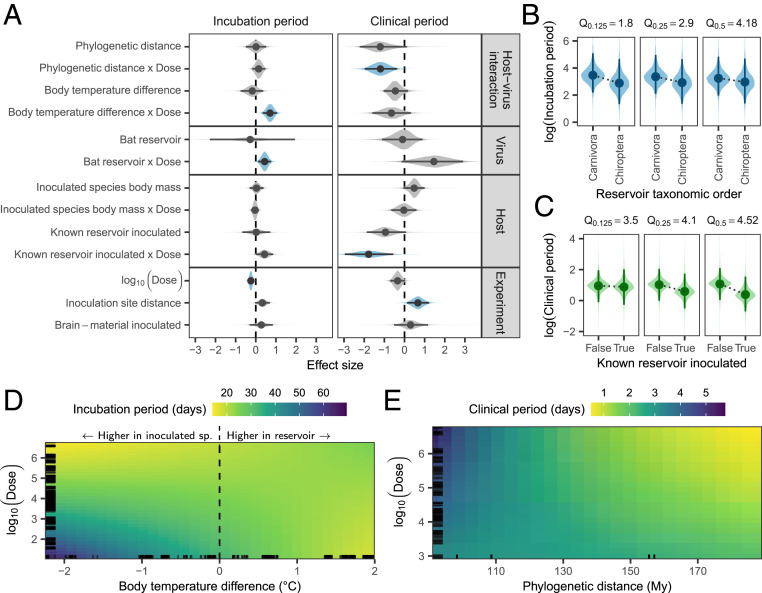
Factors affecting the progression of disease following cross-species inoculations. (*A*) Coefficient estimates from independent models fit to the duration of incubation (*n* = 443 inoculations) and clinical periods (*n* = 178 inoculations). In addition to the variables shown, both models contained corrections for nonindependence between observations from the same experiment and for inoculated species phylogeny. The regression on incubation period durations additionally contained corrections for reservoir phylogeny. Lines indicate the extent of the 95% HPD, while points indicate the posterior median. Shaded areas show the posterior distribution, with color used to indicate estimates whose 95% HPD excludes zero. (*B*–*E*) Predicted incubation period and clinical period durations when varying dose along with (*B*) reservoir taxonomic order, (*C*) whether or not the inoculated species was a known reservoir of rabies virus, (*D*) the difference in typical body temperatures between the virus reservoir and the inoculated species (specifically reservoir temperature minus inoculated species temperature), or (*E*) phylogenetic distance between species. For each set of predictions, all other explanatory variables in the model were held constant at their median observed value. In *B* and *C*, each subpanel shows the predicted effect at different quantiles, Q, of observed doses (in log_10_ mouse LD_50_), indicated above the panel. In *D* and *E*, panels are zoomed into the area containing 90% of observed values on each axis, and colors show the posterior median of predictions. Black lines along the edges of *D* and *E* indicate the locations of observed values for the variable on each axis.

We modeled incubation period duration using log-normal generalized linear mixed models (GLMMs), correcting for phylogenetic nonindependence among inoculated species and among reservoir species, as well as for clustering within experiments (*Methods*). As expected, incubation periods were shortened by both increased dose (Fig. 2*A*) and potentially also by inoculation sites which were relatively closer to the brain (95% highest posterior density interval [HPD]: −0.024 to 0.704; Fig. 2*A*). More importantly, the duration of incubation periods was also influenced by features of the virus reservoir as well as its interaction with the inoculated host. Specifically, differences in incubation period duration were associated with reservoir type (bat vs. carnivore) and with body temperature differences between source and inoculated hosts (Fig. 2*A*). Both effects depended on viral dose (HPD: 0.113 to 0.752 and 0.346 to 1.045, respectively; Fig. 2*A*). At low doses, viruses from bat reservoirs were associated with shorter incubation periods compared to viruses from carnivores, though this effect diminished at higher doses (Fig. 2*B*). There was some evidence that inoculated species which are known to be capable of acting as rabies reservoirs in nature experienced shorter incubation periods than other species at low doses (HPD: −0.002 to 0.864; Fig. 2*A*).

The difference in typical body temperature between the virus reservoir and the inoculated species had a more marked effect on incubation periods (Fig. 2*A*). The onset of symptoms was delayed when the virus was inoculated into species with a warmer body temperature than its reservoir (negative values in Fig. 2*D*), although this delay reduced with higher doses. The opposite was also true—hosts with lower body temperatures than the virus reservoir tended to have shorter incubation periods (Fig. 2*D* and *SI Appendix*, Fig. S1). Models fitting effects for inoculated and reservoir species body temperature separately allowed us to explore this temperature effect further. Regardless of viral origin, inoculated species with higher typical body temperatures tended to have longer incubation periods (interacting with dose, HPD: −1.013 to −0.071; *SI Appendix*, Fig. S2). However, viruses from reservoirs with higher body temperatures were associated with shorter incubation periods across all inoculated hosts (again interacting with dose, HPD: 0.117 to 0.588), suggesting that these viruses had adapted to counteract any losses in efficiency caused by the body temperature of their reservoir host. Many bat species have lower body temperatures than most carnivore species, but the distributions of body temperatures across bats and carnivores show considerable overlap (*SI Appendix*, Fig. S3). Importantly, there was little correlation between phylogenetic distance and body temperature difference among species (*SI Appendix*, Fig. S3), indicating that the observed temperature effects were not explainable by the level of taxonomic relatedness among species. All fixed effects combined explained 19.2% of the variation in incubation period durations (HPD: 2.8 to 40.2%).

### Clinical Period.

Once symptoms appeared, the median time to death (the clinical period) was 2 d, ranging from <1 d to 8 d. We modeled clinical period duration using log-normal GLMMs, correcting for phylogenetic clustering among inoculated species and for clustering within experiments. As observed for incubation periods, increasing relative distance between the inoculation site and the brain increased clinical period durations (HPD: 0.131 to 1.221), while dose interacted with a range of other factors (Fig. 2*A*). Cross-species inoculations between phylogenetically more distant species were associated with an increased sensitivity to high viral doses, resulting in shorter clinical periods (HPD: −1.966 to −0.363; Fig. 2 *A* and *E*). Bat-associated viruses appeared to have shorter clinical periods, and—as also observed for incubation periods—this effect depended on dose, but here it was poorly estimated (HPD: −0.144 to 2.913; Fig. 2*A*). Similarly, the clinical period durations of species which are known reservoirs responded more strongly to increasing doses than those of other species, which might be indicative of more efficient viral replication and/or cell-to-cell spread in these species (HPD: −2.958 to −0.554; Fig. 2 *A* and *C*). Combined, the fixed effects explained 43.7% of the variation in clinical period duration (HPD: 15.7 to 62.7%).

### Virus Titer in Salivary Glands.

**Fig. 3.**
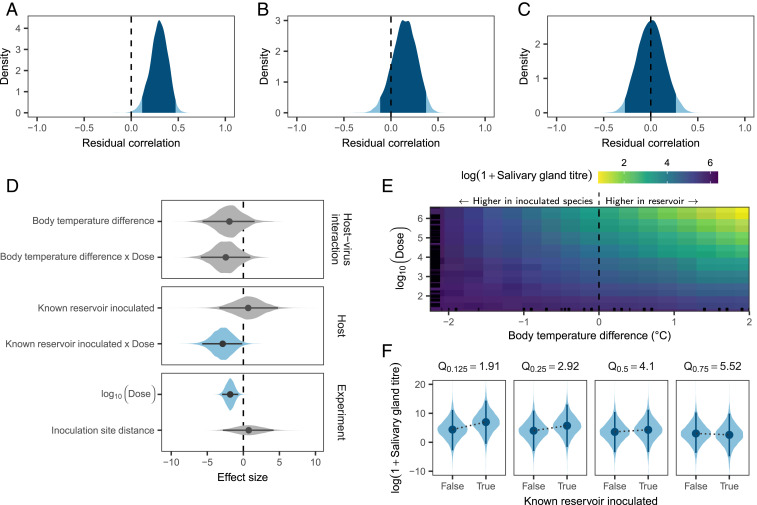
Factors predicting the titer of virus in salivary glands following cross-species inoculation (all models fitted to data from 514 inoculations, of which 278 provided data on salivary gland titers, 443 provided data on incubation period duration, and 207 included data on both). (*A*) Correlation between virus titer and the duration of incubation periods, after accounting for clustering within experiments. Shown is the estimated posterior distribution from a multiresponse regression fitted using the mcmcGLMM library in R, with the darker shaded area indicating the extent of the 95% HPD. (*B* and *C*) The remaining correlation is reduced when also accounting for correlation of virus titer and incubation period duration within the inoculated species phylogeny (*B*) and further reduced to ∼0 when additionally accounting for differences in inoculated dose (*C*). (*D*) Coefficient estimates for the regression on log virus titers in the salivary glands, when accounting for clustering within experiments and the inoculated species phylogeny. Lines indicate the extent of the 95% HPD, while points show the posterior median and shaded areas the shape of the posterior distribution, with color used to indicate estimates whose 95% HPD excludes zero. (*E* and *F*) Predicted salivary gland titers when varying dose and either the difference in body temperatures between the virus reservoir and the inoculated species (*E*) or whether or not the inoculated species is a known rabies virus reservoir (*F*), while keeping all other variables in the model constant. *E* shows the posterior median of predicted virus titers, with black lines along the edges indicating the locations of observed values and is zoomed to the area covering 90% of observed values along both axes. In *F*, predictions are shown at different quantiles of dose (in log_10_ mouse LD_50_), as indicated above each subpanel.

Onward transmission of rabies virus, which is mediated by an animal bite, requires presence of the virus in sufficiently high titers in the salivary glands. To test how the host–virus context of cross-species transmission affects the amount of virus excreted, we investigated the virus titer detected in salivary glands postmortem as a proxy. To simultaneously investigate potential explanations for the previously reported correlation between salivary gland virus titer and incubation period duration (14–16), we modeled the virus titer excreted jointly with incubation periods using a multiresponse log-normal GLMM. When accounting only for clustering within experiments, salivary gland titers showed a moderate positive correlation with incubation period duration (Pearson correlation: 0.298, HPD: 0.115 to 0.471; Fig. 3*A*). Thus, consistent with previous work, animals which experienced longer incubation periods tended to have more virus in their salivary glands postmortem. Part of this correlation is accounted for by the inoculated species phylogeny, with related species having similar incubation periods and excreting similar amounts of virus (Fig. 3*B*). The remaining residual correlation is explained by differences in dose (Fig. 3*C*), with higher doses leading to decreased salivary gland titers (Fig. 3*D*). We did not find convincing evidence for a similar correlation between clinical period durations and salivary gland titers (Pearson correlation: −0.123, HPD: −0.411 to 0.172 once correlation within experiments was accounted for), but this was based on limited data (*n* = 80). The dose-dependent body temperature difference effect observed for incubation periods was poorly estimated in the salivary gland titer model, with a large posterior median effect size (−2.445) but high levels of uncertainty (HPD: −5.896 to 0.961; Fig. 3 *D* and *E*). A somewhat clearer effect was observed for the interaction of reservoir status and dose: At low doses, known rabies reservoir species produced higher virus titers in the salivary glands than nonreservoirs (HPD: −5.698 to −0.164; Fig. 3 *G* and *F*). At very high doses, however, we detected no difference in salivary gland titers, possibly because animals succumb too fast for any differences to develop (Fig. 3*F*).

## Discussion

The progression of viral infections within the index host following cross-species transmission is a crucial determinant of onward transmission but is generally unobservable in nature. By analyzing a unique dataset of experimental cross-species infections, we demonstrate that phylogenetic distance and specific physiological differences between the host species involved alter the progression of infections in ways that are expected to influence whether further transmission occurs in the novel host.

The association between incubation period duration and the amount of virus detected in the salivary glands suggests a direct mechanism linking longer incubation periods to onward transmission. Species with higher body temperatures than the reservoir host tended to have longer incubation periods, specifically at lower viral inoculation doses (Fig. 2*D*). Although one might expect body temperature to be a phylogenetically conserved trait, we found little correlation with phylogenetic distance (*SI Appendix*, Fig. S3*A*), and others have shown that body temperatures are clustered primarily at higher taxonomic levels (25). The finding that incubation period duration is influenced by body temperature is consistent with in vitro experiments showing that temperature can affect the infectivity of rabies virus, possibly by altering the rate of cell-to-cell spread (29). Further, exposing rabies-inoculated mice to high ambient temperatures has been shown to delay the onset of symptoms (30). However, the specific mechanisms that could shorten incubation periods in a novel host environment which is colder than the host environment to which the virus is adapted as observed here remain to be identified, and we cannot exclude the possibility that body temperature differences are a proxy for other physiological differences between species.

Crucially, we found clear evidence for virus adaptation to a specific host environment (*SI Appendix*, Fig. S2), consistent with infection progression being matched to each host species. Given this host adaptation and poor correspondence between body temperature differences and phylogenetic distance, the observed temperature effect may help explain rabies virus host shifts across large phylogenetic distances. For example, despite host shifts from bats into carnivores being generally very rare, rabies virus has shifted repeatedly from big brown bats (*Eptesicus fuscus*, 36 °C) to striped skunks [*Mephitis mephitis*, 36.45 °C (12, 31)]. More generally, our results suggest that transmissions to species with warmer typical body temperatures than the current reservoir are more likely to become established, since this would be expected to result in longer incubation periods and higher virus excretion. This might explain observations suggesting sustained transmission of rabies virus lineages associated with common vampire bats (*Desmodus rotundus*, 35 °C) in sympatric frugivorous bats [*Artibeus lituratus*, 37.3 °C (31–33)].

The observation that low doses of viruses from bat reservoirs resulted in shorter incubation periods relative to those from carnivores (Fig. 2 *A* and *B*) suggests increased infectivity and/or faster within-host spread among bat-adapted rabies viruses. Since our data were limited to viruses from two bat reservoirs—with 82% of these associated with one species, *Tadarida brasiliensis* (Fig. 1)—it remains unclear whether this is a general feature of bat-associated rabies viruses. However, similar results have been observed in humans, where both incubation and clinical periods were shorter when the virus originated from bats rather than carnivores (22). This bat-associated effect may be the result of body temperature differences—at 35 and 36 °C, the two bat reservoirs included had cooler body temperatures than almost all inoculated species (*SI Appendix*, Fig. S3). Alternatively, since bats are considerably smaller than known carnivore reservoirs and likely transfer much smaller volumes of saliva during transmission, bat-associated rabies viruses may be adapted to transmit at lower doses. Although it may be expected that smaller animals would succumb faster to a given dose, we found no evidence that the interaction between the body mass of inoculated species and dose affects the duration of incubation or clinical periods (Fig. 2*A*).

Following the incubation period, the appearance of clinical signs of disease typically coincides with viral excretion and transmission. The duration of clinical signs is therefore crucial in determining whether an index host can transmit to conspecifics. Notably in the case of rabies virus, the clinical period coincides with the onset of signs such as aggression that facilitate onward spread through biting. It is also relatively short and invariably ends in death of the infected host, terminating transmission opportunities (18). Increased phylogenetic distances between virus reservoirs and inoculated species appear to reduce the duration of clinical periods (Fig. 2*E*). Such increased virulence would mean that onward transmission becomes increasingly unlikely following cross-species infection between more distant relatives. This is consistent with previous work showing that the number of successful rabies virus host shifts among North American bats decreases with phylogenetic distance (13).

Because rabies virus is generally transmitted via bite, the amount of virus excreted in the salivary glands will affect the probability of transmission from the index case. Further, the overall strong effects of dose we observed suggest that the amount of virus transferred to secondary cases will be a primary determinant of disease progression in secondary cases and hence further transmission in the new host population. Such effects on onward transmission are likely to be nonlinear, with a potentially diminishing effect at very high doses. However, it is notable that nonreservoirs tended to have lower virus titers in their salivary glands than established rabies virus reservoirs (Fig. 3 *D* and *F*), which may explain why the virus remains restricted to a relatively small number of reservoir hosts despite frequent spillovers to other species (11). Our results also provide tentative evidence of shorter incubation periods in nonreservoirs (Fig. 2*A*). The exact mechanisms underlying these differences between known rabies virus reservoirs and other species remain unexplained but are likely to be evolutionarily conserved, given the phylogenetic clustering in excretion levels apparent in our analysis.

Overall, our results point to adaptation of the rate of disease progression to match individual host species. Studies of rabies virus host shifts have thus far failed to find sites in the virus genome which consistently change during host adaptation (12, 20, 34). This has led to the suggestion that host adaptation can be achieved through numerous sets of molecular changes (34), which would indeed be the case if the requirement is to balance disease progression to the point where onward transmission becomes likely. In some host–pathogen systems, such adaptation is explained by a trade-off between selection for faster growth to maximize viral load, and thereby infectiousness, and selection for reduced host damage (virulence) to maximize transmission opportunities (35, 36). In contrast, we observed a positive correlation between salivary gland titers and incubation period duration, implying that slower host damage leads to greater infectiousness. This, in turn, suggests a trade-off between faster replication and/or spread (speeding up disease progression) and the ability to reach the salivary glands via functioning neural pathways. Such a trade-off is supported by the observation that the correlation between incubation period duration and salivary gland virus titer was modulated in part by dose (Fig. 3*C*), with higher amounts of virus reducing both.

The experimental data analyzed here offer a unique view on index infection dynamics following cross-species transmission. By revealing the complex links between dose, physiological differences between hosts, disease progression, and virus excretion, our analyses bring us closer to being able to model and predict the process of disease emergence and host shifts. The large dataset of controlled infections further enabled us to generate disease progression parameter distributions for all observed combinations of within- and cross-species transmissions which can be directly applied in future efforts to model rabies transmission dynamics (37). It is of note that some effects observed here were eventually overcome by high doses (Figs. 2 *B* and *D* and 3*F*; the median dose across all experiments was 12,679 mouse median lethal doses [LD_50_]), and future infection studies should aim to utilize doses closer to those of natural exposures. Following cross-species transmission, rabies virus shows increased virulence (i.e., more rapid death) in more distantly related species, to the point that opportunities for transmission are likely to be markedly reduced. At the same time, a mismatch in host physiological features (including features not strongly correlated with phylogeny, such as body temperature) can alter both infectivity and disease progression, with implications for onward transmission. Thus, the picture that emerges is one of a potential virulence mismatch in index infections, that may partially explain why—despite having the ability to infect all mammals and frequent involvement in cross-species transmission events—rabies virus remains restricted to a relatively small number of species-specific maintenance cycles.

While the determinants of cross-species transmission have been the subject of intense research (reviewed in refs. 5–7), the very next step, that is, what happens during the initial infection to determine the likelihood of onward transmission, has remained relatively unexplored. Our results show that meta-analyses of cross-species infection experiments provide a tractable means of investigating this process. Expanding such analyses to other viruses may allow us to identify general rules which predict the outcome of cross-species transmissions. More work is needed to understand the host features that affect the probability of infection upon exposure, the within-host mechanisms driving virulence, and the epidemiological consequences of differences in disease progression and virus excretion. Our findings illustrate how understanding these mechanisms will be key to predicting which cross-species exposures are most likely to lead to future host shifts of rabies virus, and of zoonotic diseases more broadly.

## Methods

### Literature Search and Data Collection.

A search for published rabies virus infection studies was performed as described in *SI Appendix*, *Supplementary Information Text*. Searching across the PubMed and Web of Science databases yielded 2,279 records on 16 January 2015. These records were reviewed according to the criteria listed in *SI Appendix*, Table S2 to select studies for inclusion in the meta-analysis.

From each study, we recorded individual-level data on the species inoculated, the dose, inoculation route and site, and the reservoir host species of the virus used. Response variables, when available, included the observed incubation and clinical period durations and the titer of virus present in the salivary glands postmortem. Incubation and clinical period data comprised a mixture of exact times, interval-censored times (i.e., studies only reported ranges for groups of animals), and right-censored observations (i.e., deaths unrelated to rabies before the conclusion of the study or killing of survivors at the end of each study). Right-censored observations were rare, however, and the final dataset of cross-species inoculations analyzed here contained only exact and interval-censored observations. In particular, all animals for which clinical period durations were available had been observed until rabies-induced death and with only one exception stem from studies published between 1958 and 1995 (i.e., generally from before killing of animals showing signs of disease became common practice).

Taxonomic classifications were updated to match ref. 38, by matching the scientific and common names given in each publication against the Integrated Taxonomic Information System database (https://www.itis.gov/). Data from our meta-analysis were supplemented with species-level data from the PanTHERIA database, along with body-temperature data from the AnAge database (31, 39). Because not all records were resolved to subspecies level, and external data sources only contained data at the species level, information on the specific subspecies involved was ignored in the analyses described here. This resulted in two pairs of subspecies being clustered together, while two domesticated species were analyzed using species-level data for their wild ancestor (*SI Appendix*, Table S1). Further data cleaning and validation steps are described in *SI Appendix*, *Supplementary Information Text*.

### Accelerated Failure Time Model.

The durations of incubation and clinical periods were modeled using independent GLMMs on the censored event times, in this context more frequently termed accelerated failure time models. These models perform a regression on the waiting time to some specific event (e.g., the appearance of clinical signs, signifying the end of the incubation period), with coefficients acting to increase or decrease the time to the event. We assumed a log-normal distribution for the event times T.

Thus, the duration of the incubation period of each individual i of species r (the inoculated species), inoculated with a virus from species d (the source or reservoir species) in experiment j was modeled as

$$\begin{array}{l} f\left(Y_{i,d,r,j}|\mu_{i,d,r,j},\sigma_{\epsilon }\right)∼N\left(\mu_{i,d,r,j},\sigma_{\epsilon }^{2}\right)\\ \mu_{i,d,r,j}=X_{i}^{'}\beta +p_{d}+q_{r}+m_{d}+n_{r}+o_{j}, \end{array}$$

where Y=log(T), while $\mu_{i,d,r,j}$ and $\sigma_{\epsilon }$ are the mean and SD of a normal distribution, respectively. Coefficients are represented by $\beta_{i}$, with $X_{i}^{'}$ representing a vector of data on potential explanatory variables. Finally, (p,q) and (m,n,o) respectively represent phylogenetic and nonphylogenetic random effects for the source species (the virus reservoir, p and m), inoculated species (q and n), and experiment (o).

Phylogenetic random effects were drawn from a multivariate normal distribution taking the form

$$\begin{array}{rr} p & ∼N_{k}\left(0,\sigma_{p}^{2}A_{p}\right)\\ q & ∼N_{l}\left(0,\sigma_{q}^{2}A_{q}\right), \end{array}$$

where **0** is a vector of zeros (of length k or l, equal to the number of source or inoculated species, respectively), and $\sigma_{p}^{2}$ and $\sigma_{q}^{2}$ are variance parameters. $A_{p}$ and $A_{q}$ represent correlation matrixes for all source and inoculated species, respectively. These matrixes were calculated from a composite time-scaled phylogeny generated by timetree.org (40) assuming a Brownian model of trait evolution using version 3.5 of the APE package in R (41, 42). These random effects adjust for potential correlation in the response variables due to relatedness. Similar results were obtained when using the mammalian supertree of ref. 43, but this supertree had a slightly lower resolution than the timetree.org phylogeny.

The nonphylogenetic random effects took the form

$$\begin{array}{rr} m_{d} & ∼N\left(0,\sigma_{m}^{2}\right)\\ n_{r} & ∼N\left(0,\sigma_{n}^{2}\right)\\ o_{j} & ∼N\left(0,\sigma_{o}^{2}\right), \end{array}$$

where $\sigma_{m}^{2}$ and $\sigma_{n}^{2}$, respectively, measure the variance between source and inoculated species not captured by the Brownian model (19), while $\sigma_{o}^{2}$ measures the variance between experiments. A similar model was used for the duration of clinical periods, except that data were pooled across virus reservoirs by removing the random effects for reservoir species and reservoir phylogeny (p and m above). This was necessary because the clinical period data involved viruses from just four reservoir species, making it impossible to accurately estimate the variance between observations associated with different reservoirs.

To accommodate censoring, the vector of event times, T, was treated as a latent variable. When only the range of incubation or clinical period durations was given for a specific group of animals, data were treated as interval censored, that is, $T_{i,d,r,j}\in \left[L_{i,d,r,j},U_{i,d,r,j}\right]$, where L and U represent the lower and upper boundaries of observed event times (*SI Appendix*, *Supplementary Information Text*). Exact observations were recorded by setting $L_{i,d,r,j}=U_{i,d,r,j}$.

### Multiresponse Models.

In an independent model, the amount of virus detected in salivary glands postmortem was modeled jointly with incubation period durations, to allow estimation of the amount of residual correlation between incubation period duration and the amount of virus in the salivary glands. Several authors have noted a link between these measures (14–16), but it remains unexplained.

This regression was similar to the model above, except that the normal distribution on log(observations) was replaced with a multivariate normal distribution. Thus, in the full model, the observed value Y of response variable v for individual i of species r in experiment j was modeled as

$$\begin{array}{l} f\left(Y_{v,i,r,j}|\mu_{v,i,r,j},\sigma_{\epsilon }\right)∼N_{2}\left(\mu_{v,i,r,j},\sigma_{\epsilon }\right)\\ \mu_{v,i,r,j}=X_{i}^{'}\beta_{v}+q_{v,r}+o_{v,j}, \end{array}$$

where v=1 represents the incubation period [i.e., $Y_{v=1}=\text{log}\left(T\right)$] and v=2 is the virus titer in the salivary glands. Virus titer was thus also modeled as log-normal, that is, $Y_{v=2}=\log\left(1+W\right)$, where W represents the observed titers, which may be 0 if no virus was detected in the salivary glands. $\beta_{v}$ is a vector of coefficients unique to each response variable, while ${\text{q}}_{v,r}$ and ${\text{o}}_{v,r}$ are random effects for inoculated species phylogeny and experiment, respectively.

In these models, $\sigma_{\varepsilon }$ is a variance-covariance matrix of the form

$$\sigma_{\varepsilon }=\left[\begin{array}{cc} \sigma_{\varepsilon ,v=1}^{2} & \sigma_{\varepsilon ,v=1,v=2}\\ \sigma_{\varepsilon ,v=1,v=2} & \sigma_{\varepsilon ,v=2}^{2} \end{array}\right],$$

where $\sigma_{\varepsilon ,v=1}^{2}$ is the residual variance in response variable 1 (incubation period durations) and $\sigma_{\varepsilon ,v=1,v=2}$ is the residual covariance between incubation period durations and salivary gland titers. From this, the Pearson correlation between incubation periods and salivary gland titers can be calculated as

$$\frac{\sigma_{\varepsilon ,v=1,v=2}}{\sigma_{\varepsilon ,v=1}^{2}\cdot \sigma_{\varepsilon ,v=2}^{2}}.$$

### Explanatory Variables.

Variables measuring differences between the reservoir and the inoculated species were included to assess the influence of previous virus adaptation on the outcome of infection in heterologous host species. These included the phylogenetic distance between the reservoir and the inoculated species, measured as patristic distances along the same composite time-scaled phylogeny generated by timetree.org used above. As above, similar results were obtained when using the mammalian supertree of ref. 43. We also included the difference in typical body temperatures between the reservoir and inoculated species, as an example of a physiological difference which does not appear to follow phylogenetic constraints (25), because temperature is known to affect rabies virus infectivity in vitro (29). Finally, a binary variable distinguishing viruses derived from bat and carnivore reservoirs was included, because differences in the clinical presentation of bat- and carnivore-associated rabies virus infection in humans have been noted (22).

Features of the inoculated host species where accommodated primarily through random effects for species and inoculated species phylogeny. However, we also included a measure of the typical body mass of the inoculated species, since larger species may be proportionally more resistant to the effects of a given dose of virus. Because only some species maintain rabies virus transmission endemically, for reasons that are not well understood, a binary variable distinguishing known reservoirs of rabies virus from other inoculated species was also included.

Differences between experiments were accommodated by including variables for dose, the inoculation site, and whether the inoculum consisted of brain material or was derived from salivary glands/saliva, along with a random effect distinguishing between experiments to accommodate any remaining differences. Because the doses encountered in these experiments differed over several orders of magnitude and the effects of increasing dose is assumed to decrease (saturate) at very large doses, this variable was included in its log-transformed form. The varying inoculation sites encountered were summarized as a “proportional inoculation distance,” representing the relative distance between the inoculation site and the brain (the primary site of rabies virus replication). This distance was calculated by classifying inoculation sites by body part (head, neck, torso, or limbs) and depth (intracranial, intramuscular, or subcutaneous) and was expressed as a proportion, where 1 indicates the furthest and shallowest possible inoculation site relative to the brain (subcutaneous inoculation of a limb), while 0 indicates intracerebral inoculation (*SI Appendix*, Table S3). Such proportional scaling means this variable is independent of the differing body sizes of the inoculated species, which allowed us to account for inoculation site and inoculated species body size in the same model. This, in turn, allowed us to include an interaction term between dose and the typical body mass of each inoculated species, to capture potential differences in dose–response between species of different sizes. Including a similar interaction between our inoculation distance measure and body mass caused identifiability issues, with the model unable to distinguish between the main effect of inoculation distance and the effect of this interaction. We therefore concluded that such an interaction was not needed here. Finally, because larger doses may compensate for any decreases in infectivity caused by features of the inoculated species and/or physiological differences between the inoculated species and the reservoir to which the virus was adapted, we also included interactions between dose and all other host and virus effects above.

### Model Fitting.

Models were fit using version 2.25 of the MCMCglmm package in R version 3.5.1 (44, 45). All coefficients and the residual variance parameter received the default prior distributions used by MCMCglmm, while parameter-expanded priors were used for the variance parameters of all random effects (46). For the incubation and clinical period duration datasets, models were fitted using 5 million MCMC steps, saving every 500th sample. Joint incubation period duration–salivary gland titer models were fitted using 1 million MCMC steps, saving every 100th sample. The first 10% of samples in each chain were discarded as burn-in. Results were inspected and summarized using version 0.18-1 of the coda package in R (47). Effective sample sizes were checked to ensure efficient sampling was achieved, and chains were visually inspected for convergence.

## Supplementary Material

Supplementary File

## Data Availability

Raw data and all data processing and analysis code have been deposited in Zenodo (https://doi.org/10.5281/zenodo.3746609).
