# Supplementary material for: Virulence mismatches in index hosts shape the outcomes of cross-species transmission
Source: Proc Natl Acad Sci U S A. 2020 Oct 29;117(46):28859–66. doi: 10.1073/pnas.2006778117 (PMC7682402; doi:10.1073/pnas.2006778117)
Supplement: Supplementary File [file pnas.2006778117.sapp.pdf]

PNAS
www.pnas.org

Supplementary Information for

Virulence mismatches in index hosts shape the outcomes of cross-species transmission

Nardus Mollentze[1,2], Daniel G. Streicker[1,2], Pablo R. Murcia[2], Katie Hampson[1] & Roman Biek[1]

[1] Institute of Biodiversity, Animal Health and Comparative Medicine, University of Glasgow, Glasgow, United Kingdom
[2] MRC – University of Glasgow Centre for Virus Research, Glasgow, United Kingdom

Corresponding author: Nardus Mollentze
Email: nardus.mollentze@glasgow.ac.uk

This PDF file includes:

Supplementary text
Figures S1 to S6
Tables S1 to S3
SI References

Other supplementary materials for this manuscript include the following:

Supplemental Dataset S1: Rabies disease progression parameters for all observed combinations of source and recipient species (including within-species inoculations)

Supplemental Dataset S2: Raw data and analysis code

Both datasets are available at https://doi.org/10.5281/zenodo.3746609

Supplementary Information Text

Literature search. To allow optimisation of the literature search strategy, a set of publications of known relevance (according to the abstract review criteria, table S2) was obtained by manual searching and from citations in several rabies textbooks. A script utilizing the BioPython library in Python 3 was used to find a search query that returned the greatest possible number of these publications in a PubMed search, whilst excluding the most common sources of false positives – notably annual veterinary reports and vaccine studies. This resulted in the following query for Web of Science:

```
TS=(rabies AND
     (inoculated OR inoculate OR
       ((intranasal* OR intracerebral* OR
         intracranial* OR intramuscular* OR
         subcutaneous* OR intraperitoneal*)
        AND (inoculat* OR challenge OR inject*) NOT vaccine) OR
       "experimental infection" OR
       "experimental rabies infection" OR
       (experiment* NEAR transmission))
     NOT (review OR "fixed virus" OR "fixed rabies virus" OR
           "annual report")
```

The same query was used to search the PubMed database, except that all instances of the NEAR operator were replaced with AND (PubMed does not support proximity searching). The PubMed version of this query returned 46 out of the 53 publications of known importance confirmed to be in the PubMed database. Of the remaining seven, five publications did not have searchable abstracts in the PubMed database.

Combining the results from Web of Science and PubMed yielded 2501 records on 16 January 2015. The search results were further filtered to exclude records where the keyword rabies did not occur in either the English title or abstract, although records where either of these fields was empty were also retained. The abstracts of the resulting 2279 records were reviewed according to the criteria in table S2. The abstracts of 35 records (1.54% of the filtered search results) could not be obtained in time for consideration. A total of 412 records (18.36% of the remaining search results) were selected for full text review, and the full texts of 382 publications (92.72%) were successfully obtained.

Publications in languages other than English were subjected to optical character recognition (where needed) using VietOCR version 4.0, a graphical user interface for the Tesseract OCR engine (version 3.30RC), optimised for each language being recognised. The digitised text was manually corrected and machine-translated using the statistical machine translation version of Google translate. All full texts were then reviewed using the criteria in table S2, resulting in the selection of 63 publications for inclusion in this study.

Data cleaning and validation. Individual-level data on all inoculated animals present in the selected publications were recorded. Following data-entry, the raw data was extensively checked for accuracy and consistency. The scientific names of all inoculated and source species were manually checked for current validity using the ITIS database and standardised to match the taxonomy of (1). A further literature search was performed to obtain data on whether each of the included species is a known maintenance host of rabies virus. Records where the source species was not a known maintenance host were validated against the original publication to check for passaging and information on the maintenance host associated with the virus inoculum used. All virus inoculum identifiers were manually checked to identify the re-use of viruses in multiple publications, and were corrected to reflect this shared origin.

Some data were excluded from the current study, but retained in the database for potential future studies. A total of 197 records were excluded because the method of titration used to determine the dose inoculated was either not known (generally negative control animals) or because their reported inoculation dose involved a tissue culture-based rather than an in vivo method of titration. All remaining records listed

virus doses obtained by intra-cerebral inoculation of mice to determine the 50% lethal dose. A further 31 records with a reported dose of 0, representing non-inoculated control animals, were also excluded. A total of 131 records involving five source species were excluded because the source species was either not a known maintenance host of rabies virus or could not be resolved to the species level, and 17 records involving one inoculated species were excluded because the inoculated species could not be resolved to species level.

A derived variable was created to unify diverse descriptions of inoculation routes and sites, based on the proportional distance of each inoculation site from the brain. Inoculation sites were classified into four zones on the body and three depths, and distances were assigned to each category (table S3). These distances were then normalised to lie in [0,1]. We assumed that sub-cutaneous inoculation of the head was equivalent to intramuscular inoculation of the neck. Further, relative to the assumption of equal spacing between sites, our assumed distances place the neck and torso region closer to the brain, and limbs correspondingly further (figure S4). This was done to reflect the fact that inoculation sites in the neck and torso are closer to the spinal cord. Inoculation sites that were not specific enough to be classified into this scheme were checked against the original publications, and 115 records where the inoculation site information could not be improved were excluded.

All available data on the timing of disease progression and the total number of days survived following inoculation were recorded. When timing data was reported for groups of animals, the event times for each animal in the group was recorded as known only to be within the interval reported for that group (a form of interval censoring). Data on the total number of days survived was recorded in both exact ($\boldsymbol{T}_{\text{exact}}$) and interval censored ($\boldsymbol{T}_{\text{lower}}$ and $\boldsymbol{T}_{\text{upper}}$) forms, while the length of incubation and clinical periods were recorded in interval censored form only (with both columns given the same value when an exact time was available). Although only two of these timing variables needs to be known to calculate the third, data was recorded only in the form reported in each study.

The data were then processed as described in figures S5 – S6, and timing data were checked for internal consistency. This involved consolidating information on the cause of death, results of clinical observations and post-mortem diagnostic tests and the timing of disease progression stages associated with each animal into variables describing the lower and upper limits of the incubation and clinical period duration. When rabies was confirmed for a given animal through either clinical diagnosis or post-mortem diagnostic tests, it was assumed to have rabies. In many cases, data could be calculated for unreported timing variables by using some combination of reported data on either the number of days survived post inoculation, the duration of the incubation period, and/or the duration of the clinical period. For example, if only the earliest day that an animal $\boldsymbol{i}$ could have died ($\boldsymbol{T}_{\boldsymbol{i},\text{lower}}$) as well as the upper limit of the clinical period for that animal ($\boldsymbol{M}_{\boldsymbol{i},\text{upper}}$) is known, the lower limit of the incubation period can be calculated as $\boldsymbol{I}_{\boldsymbol{i},\text{lower}} = \boldsymbol{T}_{\boldsymbol{i},\text{lower}} - \boldsymbol{M}_{\boldsymbol{i},\text{upper}}$ (figure S5).

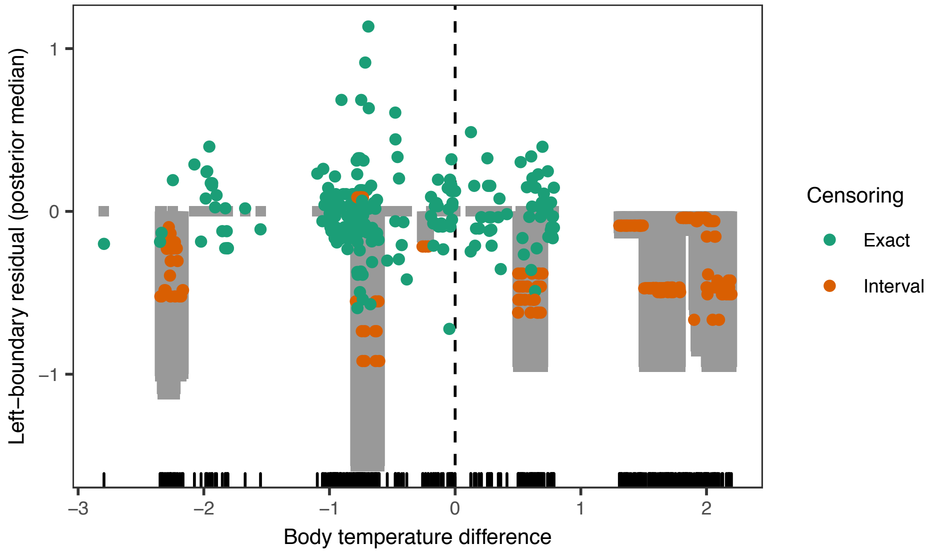

Fig. S1. Posterior median residuals as a function of body temperature difference. The observed data was subject to censoring, but the lower-boundary was always known. Residuals were calculated as the observed lower limit minus the fitted value, with grey-shaded areas showing the expected range of each observation. In the case of exact observations, a perfect fit would result in residuals equal to 0, while the residuals for interval-censored observations are expected to lie in [0, lower - upper). No systematic bias in residuals was observed, supporting the conclusion that the effect of body temperature distance remained linear for both positive and negative values.

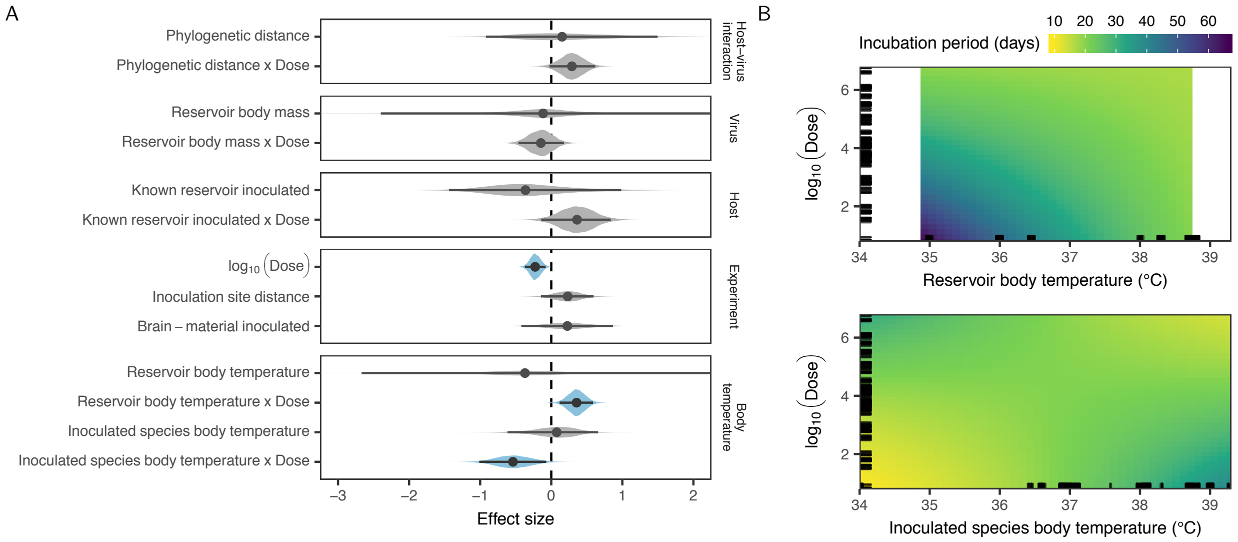

Fig. S2. Coefficient estimates and predicted effects when fitting separate effects for the typical body temperature of the inoculated species and virus reservoir. (A) Coefficient estimates, with lines showing the 95% highest posterior distribution and points showing the posterior median. Compared to the model shown in the main text, this model does not contain an effect for inoculated species body mass (which had no effect in the full model, and is correlated with inoculated species body temperature) and contains an effect for reservoir body mass, replacing the variable separating bat-associated viruses from carnivore-associated viruses (which was correlated with inoculated species phylogeny). (B) Posterior median predicted incubation periods as a function of reservoir (top) or inoculated species body temperature (bottom) and dose. Predictions are shown within the range of observed body temperature values in the dataset, and over the range covering 90% of observed doses.

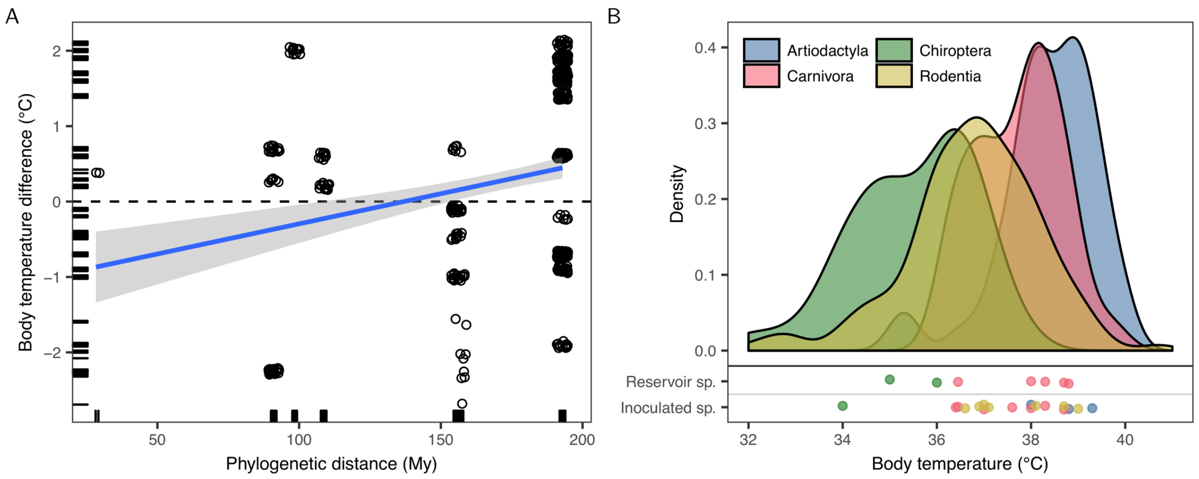

Fig. S3. Relationship between phylogenetic distance and host body temperature. (A) Relationship between phylogenetic distance (in millions of years) and body temperature difference in the incubation period dataset. Points are shown jittered for clarity, while the blue line represents the best linear fit (adjusted R^2 = 0.049, $p < 0.001$). (B) Distribution of body temperature in the taxonomic orders of hosts included in this study, based on all species for which body temperature data are available in the AnAge database. Points indicate the body temperatures for species included in the incubation period dataset as either reservoirs or inoculated species, jittered vertically to reduce overlap.

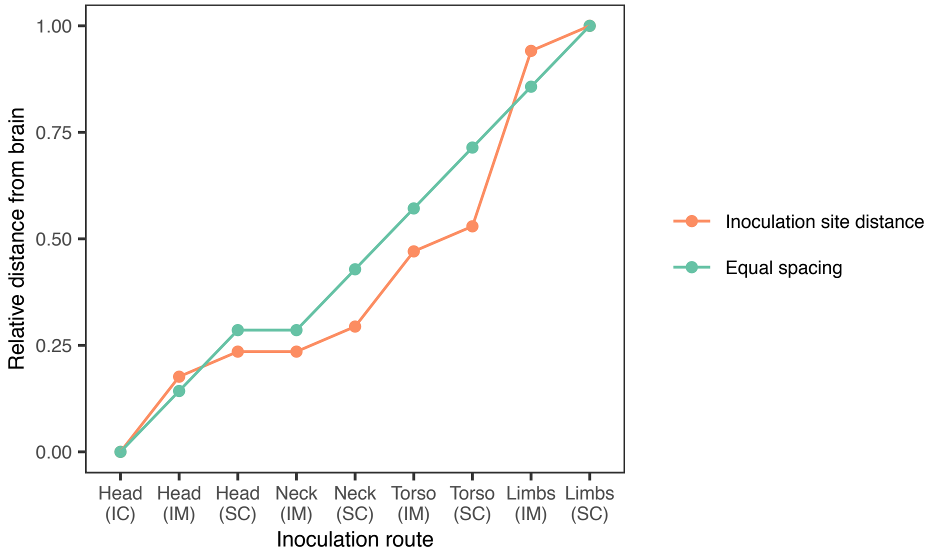

Fig. S4. Assumed distances between inoculation sites, compared to the simpler assumption of equal spacing between sites. IC = intracranial, IM = intramuscular, and SC = subcutaneous inoculation.

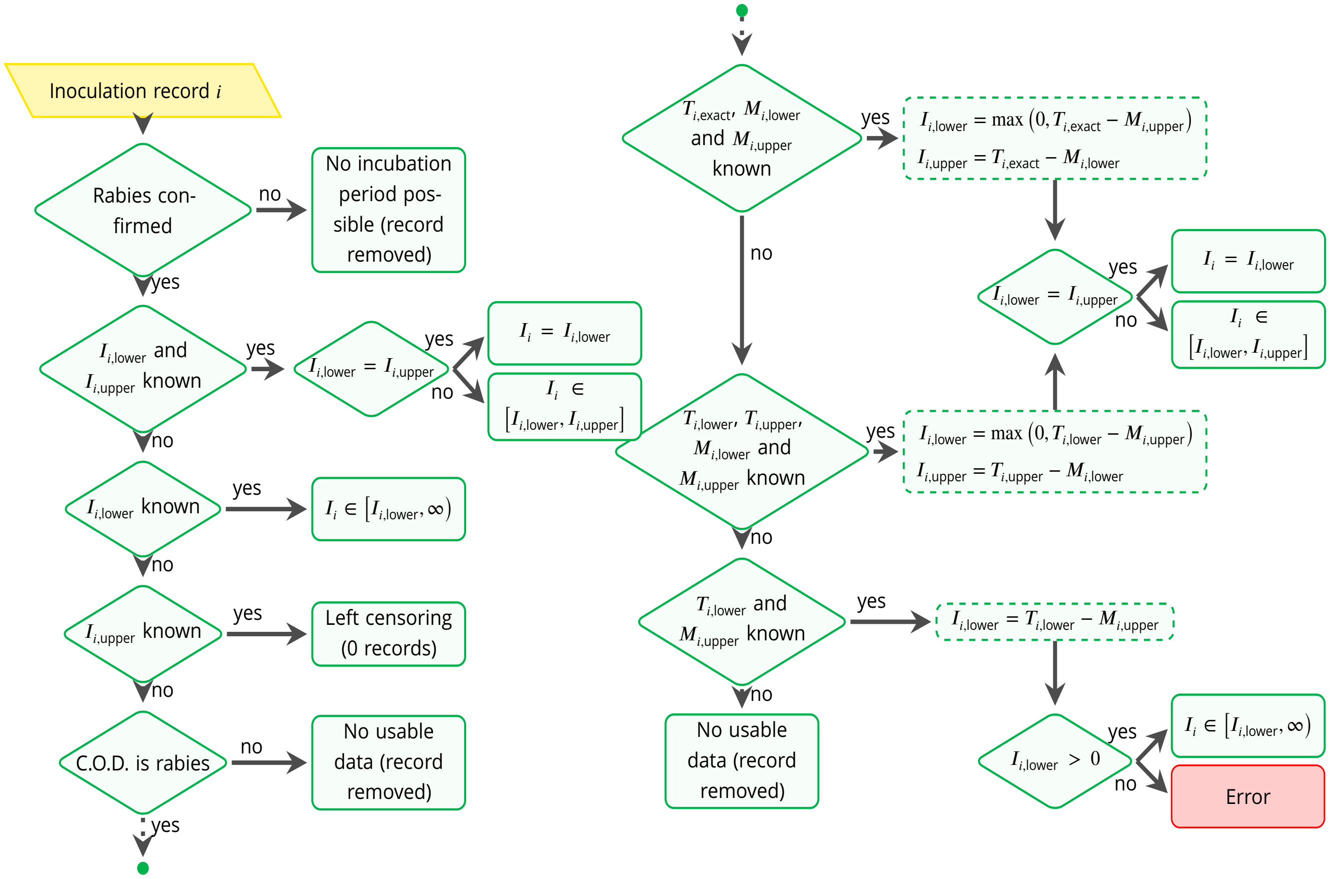

Fig. S5. Overview of the algorithm used to consolidate data on incubation periods.

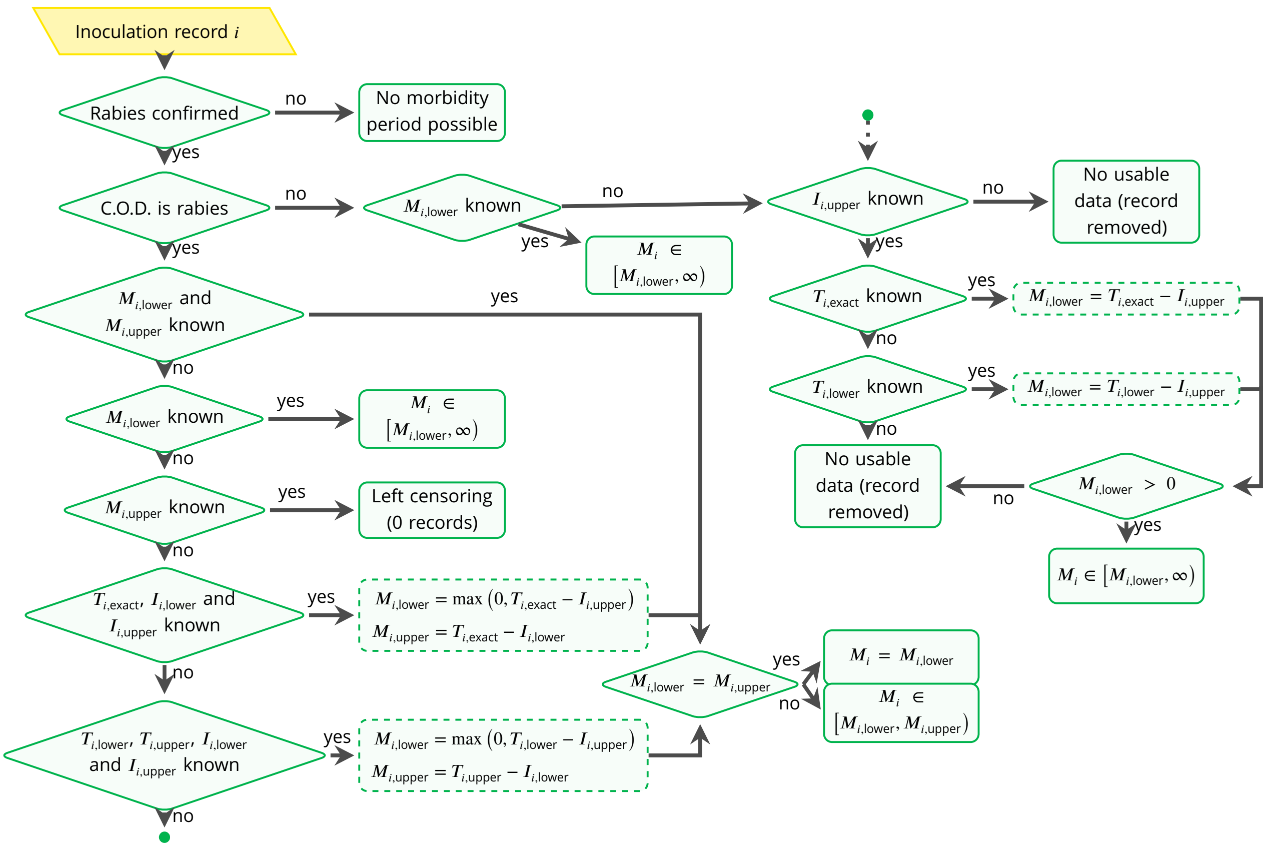

Fig. S6: Overview of the algorithm used to consolidate data on clinical periods.

Table S1. Known sub-species in the dataset, which were analysed at the species level

Species	Subspecies	Common name
Antrozous pallidus	*pallidus*	Pallid bat
Canis lupus	*familiaris*	Domestic dog
Mustela putorius	*furo*	Domestic ferret
Tadarida brasiliensis	*mexicana*	Mexican free-tailed bat
	brasiliensis	Brazilian free-tailed bat
Vulpes vulpes	*crucigera*	European red fox
	fulves	Eastern North American red fox

Table S2. Criteria used to select publications.

Review stage	Criteria
Abstract review	Involves experimental inoculations with rabies virus
	Does not solely focus on laboratory-adapted virus strain
Full text review	Source and inoculated species clearly identified
	Source host species is a known maintenance host of rabies virus (passaging in non-maintenance hosts allowed)
	Inoculated host species is a mammal
	Inoculated host species is wild-type or outbred
	Inoculated dose stated (in mouse LD_{50})
	Healthy, unvaccinated animals used
	Multiple experimental groups or a control group present

Table S3. Distances assigned to each inoculation site category to express relative distance to the brain*

	**Intracranial (0)**	**Intramuscular (1/7)**	**Subcutaneous (3/14)**
**Head (1/14)**	0	1/14 + 1/7	1/14 + 3/14
**Neck (1/7)**	-	1/7 + 1/7	1/7 + 3/14
**Torso (3/7)**	-	3/7 + 1/7	3/7 + 3/14
**Limbs (7/7)**	-	7/7 + 1/7	7/7 + 3/14

*Distances were chosen to ensure intramuscular inoculations to the neck received the same value as subcutaneous inoculations to the head. This leaves 7 distinct inoculation sites on the body, plus intracranial inoculations. All distances were subsequently scaled to range between 0 (intracranial, head) and 1 (subcutaneous, limbs).

SI References

1. D. E. Wilson, D. M. Reeder, Eds., *Mammal species of the world: a taxonomic and geographic reference*, 3rd Ed. (Johns Hopkins University Press, 2005).
